# Fractional Langevin model of gait variability

**DOI:** 10.1186/1743-0003-2-24

**Published:** 2005-08-02

**Authors:** Bruce J West, Miroslaw Latka

**Affiliations:** 1 Mathematical and Informational Sciences Directorate US Army Research Office, P.O. Box 12211 Research Triangle Park, NC 27709, USA; 2 Physics Department Wroclaw University of Technology Wybrzeze Wyspianskiego 27, 50-370 Wroclaw, Poland

## Abstract

The stride interval in healthy human gait fluctuates from step to step in a random manner and scaling of the interstride interval time series motivated previous investigators to conclude that this time series is fractal. Early studies suggested that gait is a monofractal process, but more recent work indicates the time series is weakly multifractal. Herein we present additional evidence for the weakly multifractal nature of gait. We use the stride interval time series obtained from ten healthy adults walking at a normal relaxed pace for approximately fifteen minutes each as our data set. A fractional Langevin equation is constructed to model the underlying motor control system in which the order of the fractional derivative is itself a stochastic quantity. Using this model we find the fractal dimension for each of the ten data sets to be in agreement with earlier analyses. However, with the present model we are able to draw additional conclusions regarding the nature of the control system guiding walking. The analysis presented herein suggests that the observed scaling in interstride interval data may not be due to long-term memory alone, but may, in fact, be due partly to the statistics.

## Background

One strategy for understanding legged locomotion of animals is through the use of a Central Pattern Generator (CPG), an intraspinal network of neurons capable of producing a syncopated output [1]. The implicit assumption in such an interpretation is that a given limb moves in direct proportion to the voltage generated in a specific part of the CPG. As Collins and Richmond [1] point out, in spite of the studies establishing the existence of a CPG in the central nervous system of quadrupeds, such direct evidence does not exist for a vertebrate CPG for legged locomotion. Consequently, these and other authors have turned to the construction of models, based on the coupling of linear and nonlinear oscillators, to establish that the mathematical models are sufficiently robust to mimic the locomotion characteristics observed in the movements of segmented bipeds [2], as well as in quadrupeds [3]. These characteristics, such as the switching among multiple gait patterns, is shown to not depend on the detailed dynamics of the constituent nonlinear oscillators, nor on their inter-oscillator coupling strengths [1]. A nonlinear stochastic model of the dynamics of the human gait motor control system called the super CPG (SCPG) has been developed [4]. In the SCPG the stride interval time series is shown to be slightly multifractal, with a fractal dimension that is sensitive to physiologic stress. Herein we do not focus on the generation of each step during walking, but rather we examine the variation in successive steps and its underlying structure.

It has been known for over a century that there is a variation in the stride interval of humans during walking of approximately 3–4%. This random variability is so small that the biomechanical community has historically considered these fluctuations to be an uncorrelated random process, such as might be generated by a simple random walk. In practice this means that the fluctuations in gait were thought not to contain any useful information about the underlying motor control process. On the other hand, Hausdorff et al. [5,6] demonstrated that stride-interval time series exhibit long-time correlations, and suggested that the phenomenon of walking is a self-similar fractal activity. Subsequent studies by West and Griffin [7-9] support these conclusions using a completely different experimental protocol for generating the stride-interval time series data and very different methods of analysis. It was found that things are not quite that simple, however, and instead of the process having no characteristic time scale, as would be the case for a monofractal, there is a preference for a multiplicative time scale in the physiological control system [7].

Physiological time series invariably contain fluctuations so that when sampled *N *times the data set {*X*_*j*_}, *j *= *1,..., N*, appear to be a sequence of random points. Examples of such data are the interbeat intervals of the human heart [10,11], interstride intervals of human gait [5,9], brain wave data from EEGs [12] and interbreath intervals [13], to name a few. The analysis of the time series in each of these cases has made use of random walk concepts in both the analysis of the data and in the interpretation of the results. For example, the mean-square value of the dynamical variable in each of these cases (and many more) have the form 〈*X*(*t*)^2^〉∝ *t*^*δ*^, where *δ *≠ 1 corresponds to "anomalous diffusion". A value of *δ *< 1 is often interpreted as an antipersistent process in which a step in one direction is preferentially followed by a step reversal. A value of *δ *> 1 is often interpreted as a persistent process in which a step in one direction is preferentially followed by another step in the same direction. A value of *δ *= 1 is, again, often interpreted as ordinary diffusion in which the steps are independent of one another. The initial analysis of each of these time series, using random walk concepts, suggested that they could be interpreted as monofractals. However, on further investigation the heart beat variability has been found to be multifractal [14], as were the interstride intervals [4].

A modeling approach complementary to random walks is the Langevin equation, a stochastic equation of motion for the dynamical variables in a physical system. This latter model has undergone a transformation similar to that of random walks since its introduction into physics by Langevin in 1908. The solution to the Langevin equation is a fluctuating trajectory for the particle of interest and an ensemble of such trajectories determines the statistical distribution function. In this way the Gaussian probability density for Brownian motion is obtained. The density can also be obtained by aggregating the steps to form a discrete trajectory using a random walk model [15,16]. These two kinds of models of the physical world, random walks and the Langevin equation, have long been thought to be equivalent. In fact, that equivalence has been used as the dynamical foundation of statistical mechanics and thermodynamics. This equivalence has also been used to interpret the monofractal statistical properties of physiological time series.

While the properties of monofractals are determined by the global scaling exponent, there exists a more general class of heterogenous signals known as *multifractals *which are made up of many interwoven subsets with different *local *scaling exponents *h*. The statistical properties of these subsets may be characterized by the distribution of fractal dimensions *f(h)*. In order to describe the scaling properties of multifractal signals it is necessary to use many local Hölder exponents. Formally, the Hölder exponent *h(t*_0_*) *of a trajectory *X(t) *at *t = t*_0 _is defined as the largest exponent such that there exists a polynomial *P*_*n*_*(t) *of order *n *that satisfies the following condition [17]:



for *t *in a neighborhood of *t*_0 _and the symbol *O*(*ε*) means a term no greater than *ε*. Thus the Hölder exponent measures the singularity of a trajectory at a given point. For example, *h(t*_0_*) = 1.5 *implies that the trajectory *X *is differentiable at *t*_0 _but its derivative is not. The singularity lies in the second derivative of *X(t)*. The singularity spectrum *f(h) *of the signal may be defined as the function that for a fixed value of *h *yields the Hausdorff dimension of the set of points *t*. The singularity spectrum is used to determine whether or not the stride interval time series is multifractal.

A new kind of random walk has recently been developed, one having multifractal properties [18-21]. Herein we are guided by this earlier work, but use it to generalize the Langevin equation to describe a multifractal dynamical phenomenon. In *Methods *we review the multifractal formalism and apply the processing algorithm to the interstride interval time series. The mass exponent *τ*(*q*) is determined to be a nonlinear function of the moment *q*, and the singularity spectrum *f(h) *is found to be a convex function of local scaling exponent *h*. We also introduce a fractional Langevin equation and make the index of a fractional integral a random variable to show how this model can describe a multifractal process. The multifractal spectrum is shown to be a property of the solution to this fractional Langevin equation. In *Results and Disscussion *we apply the analytic expression for the singularity spectrum to the interstride interval data discussed in the Methods section. The agreement between the predictions of the fractional Langevin equation and experiment for human gait is remarkable. In *Conclusions *we explore some of the physiological implications of the fractional Langevin model including the suggestion that the observed scaling of the time series may not only be due to long-term memory but to the underlying statistics as well.

## Methods

The distribution of Hölder exponents for a time series can be determined in a number of different ways. Herein we use the partition function. Let us cover the time axis with cells of size *δ *such that the time is given by *t *= *Nδ *and *N > *> 1. Following Falconer [17] we can define the partition function in terms of the moments, *q*, of a measure μ



where *B*_*j *_is the *j*^*th *^box in the δ-coordinate mesh that intersect with the measure μ. We can construct the measure using the time series obtained from the interstride interval data. This measure is made by aggregating the observed interstride time intervals, *t*_*j*_, *j *= 1,2.., *N*,



such that *T(n,δ) *is interpreted as the random walk trajectory for a given data set. We use the random walk trajectory to construct the phenomenological measure in the partition function (2) as



where the integer *n *is the discrete time lag. For a monofractal random walk process the measure (4) is essentially uniform. For a multifractal, on the other hand, the theoretical scaling behavior of the partition function *S*_*q*_*(δ) *in the limit of vanishing grid scale [17,24] is

*S_q_*(δ) ≈ δ^-τ(*q*) ^    (5)

where *τ(q) *defines the mass exponent. We emphasize that (4) is a phenomenological measure with an undetermined lag time. The lag time is chosen in the present calculation to maximize the sensitivity of the partition function to the positive moments.

The mass exponent is related to the generalized dimension *D(q) *by the relation

τ(*q*) = (1 - *q*)*D*(*q*)     (6)

where *D(0) *is the fractal or box-counting dimension, *D(1) *is the information dimension and *D(2) *is the correlation dimension [24]. The moment *q *therefore accentuates different aspects of the underlying dynamical process. For *q > 0*, the partition function *S*_*q*_*(δ) *emphasizes large fluctuations and strong singularities through the generalized dimensions, whereas for *q *<*0*, the partition function stresses the small fluctuations and the weak singularities. This property of the partition function deserves a cautionary note because the negative moments can easily become unstable, introducing artifacts into the calculation. For this reason the interpretation of the trajectory approach must be judged with some caution for *q *< 0.

A monofractal time series can be characterized by a single fractal dimension. In general, time series have a local fractal exponent *h *that varies over the course of the trajectory. The function *f(h)*, called the multifractal or singularity spectrum, describes how the local fractal exponents contribute to such time series. Here *h *and *f *are independent variables, as are *q *and *τ*. The general formalism of Legendre transform pairs interrelates these two sets of variables by the relation, using the sign convention in Feder [24],

*f*(*h*) = *qh *+ τ(*q*).     (7)

The local Hölder exponent *h *varies with the *q*-dependent mass exponent through the equality



so the singularity spectrum can be written as

*f*(*h*(*q*)) = - *q*τ'(*q*) + τ(*q*)     (9)

where *τ(q) *is determined by data, that is, by the trajectory, as is its derivative *τ'(q)*.

The multifractal behavior of time series can be modeled using a number of different formalisms. For example, a random walk [19,23], in which a multiplicative coefficient in the random walk is itself made random, becomes a multifractal process. This approach was developed long before the identification of fractals and multifractals and may be found in Feller's book [25] under the heading of subordination processes. The multifractal random walks have been used to model various physiological phenomena. Another method, one that involves an integral kernel with a random parameter, was used to model turbulent fluid flow [26]. Here we adopt a version of the integral kernel, but one adapted to time rather than space series. In order to accomplish this we review some of the history of the Langevin equation.

### Fractional Langevin equation

A theoretical Langevin equation is generally constructed from a Hamiltonian model for a simple dynamical system coupled to the environment [27]. The equations of motion for the coupled system are manipulated so as to eliminate the degrees of freedom of the environment from the dynamical description of the system. Only the initial state of the environment (heat bath) remains in the Langevin description, where the random nature of the driving force is inserted through the choice of distribution of the initial states of the bath. The simplest Langevin equation for a dynamical system open to the environment has the form



where *ξ(t) *is a random process, λ is a dissipation parameter and there exists a fluctuation-dissipation relation [27] connecting the two. Of course, we cannot completely interpret (10) until we specify the statistical properties of the *ξ*-fluctuations and for this we need to know the environment of the system. The random driver is typically assumed to be a Wiener process, that is, to have Gaussian statistics and no memory.

When the system dynamics depends on what occurred earlier, that is, the environment has a memory, (10) is no longer adequate and the Langevin equation must be modified. The generalized Langevin equation takes this memory into account through an integral term of the form



where the memory kernel, *K(t)*, replaces the dissipation parameter and there is a generalized fluctuation-dissipation relation [27]. Both these Langevin equations are monofractal if the fluctuations are monofractal, which is to say the time series given by the trajectory *X(t) *is a fractal random process, if *ξ(t) *is a fractal random process.

Now we come to the most recent generalization of the Langevin equation, one that incorporates memory into the system's dynamics through the use of fractional calculus. The simplest fractional Langevin equation has the form [28]



where  is a Riemann-Liouville (*RL*) fractional derivative with 0 < β ≤ 1



and is related to the *RL*-fractional integral



Note that we have not included dissipation in this simple model, but the initial condition *X*_0 _= *X(0) *is incorporated into the dynamical equation in order to have a well-defined initial value problem. The formal solution to the fractional Langevin equation (12) is [28]



where the kernel in (15) is given by the weighting factor within the *RL*-fractional integral. As mentioned earlier, the form of this relation for multiplicative stochastic processes and its association with multifractals had been noted in the phenomenon of turbulent fluid flow [26], through a space, rather than time, integration kernel.

### Multifractal time series

The random forcing term on the right-hand side of (15) is selected to be a zero-centered, Gaussian random variable and therefore to scale as [29]

ξ(λ*t*) = λ^*h*^ξ(*t*)     (16)

where the Hölder exponent is in the range 0 <*h *= 1. In a similar way the kernel in (15) is easily shown to scale as

*K*_β_(λ*t*) = λ^β-1^*K*_β_(*t*)     (17)

so that the solution to the fractional Langevin equation scales as

Δ*X*(λ*t*) = λ^*h*+β^Δ*X*(*t*)     (18)

where Δ*X*(*t*) = *X*(*t*) - *X*_0_. In order to make the solution to the fractional Langevin equation a multifractal we assume that the parameter β is random. To construct the traditional measures of multifractal stochastic processes we calculate the *q*^*th *^moment of the solution by averaging over both the random force ξ and the random parameter β to obtain, in an obvious notation,



Note that when ζ*(q)*, the structure function exponent, is linear in *q *the underlying process is monofractal, whereas, when ζ *(q) *is nonlinear in *q *the proces is multifractal. This is the case because

ζ(*q*) = 1 - τ(*q*)     (20)

relating the structure function exponent to the mass exponent [30].

To determine the structure function exponent we make an assumption about the statistics of the parameter β. We can always write the β-average as



where *Z(s) *is a random variable as well as a function of *s*. Note that in the present case the functionality is just one of linear proportionality. In this way the expression on the right-hand side of (21) is the Laplace transform of the probability density. We assume the random variable *Z(s) *is an α-stable Lévy process in which case the statistics of the multiplicative fluctuations are given by the distribution [15]



with 0 < α = 2. Inserting (22) into (21) to replace the averaging bracket and integrating over *z *yield the delta function *δ(k+iq) *which, integrating over *k*, results in



so that re-introducing *s = lnλ *into this equation we obtain



Consequently, from (20) we obtain for the moment correlation function

ζ(*q*) = *qh *- *b*|*q*|^α ^    (23)

Therefore the solution to the fractal Langevin equation corresponds to a monofractal process only in the case α = 1 and *q *> 0, otherwise the process is multifractal. We restrict the remaining discussion to *q *> 0.

Thus, we observe that when the memory kernel in the fractional Langevin equation is random, the solution consists of the product of two random quantities giving rise to a multifractal statistical process. This is analogous to Feller's subordination process. We observe that, for the statistics of the multiplicative exponent given by Lévy statistics, the singularity spectrum as a function of the positive moments, is

*f*(*q*) = 1 - (α - 1)*bq*^α ^    (25)

which is determined by substituting (24) into (9), through the relationships between exponents (20).

## Results and Discussion

**Figure 1:**
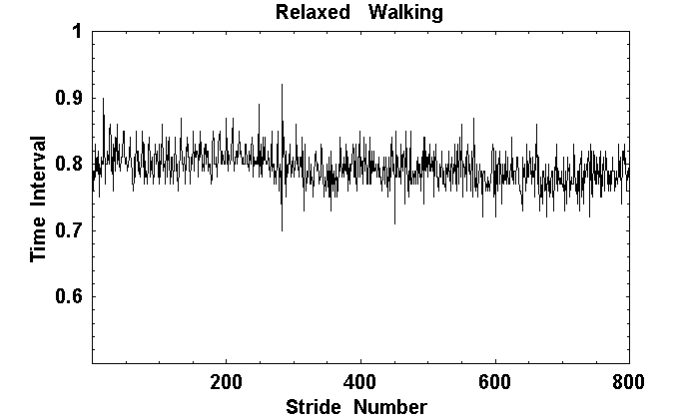
**Typical interstride interval time series: **The interstride interval time series for a person undergoing relaxed walking is depicted for 800 steps. This is taken from a 15 minute time series [7].

The data obtained, from individuals walking at a normal steady pace, consists of the time interval for each stride and the number of strides in a sequence of steps. The maximal extension of the right leg, the "stride interval" versus the stride number, plotted on a graph, has all the characteristics of a time series, *cf*. Figure 1. There were ten participants in the study (four males and six females), all in good health, with no acute injuries, ranging in age from 20 to 46 years old with a median age of 29 years. Normal steady walking was monitored for the ten participants, and an electrogoniometer was used to collect kinematic data on the angular extension of the right leg. The signal from the electrogoniometer was recorded at 100 Hz by a computer contained in a "fanny pank" attached to the walker. These data were downloaded to a PC after twelve to fifteen minutes and the interval between successive maximal extensions of the right leg in the analog signal was digitized and used as the time series data [7].

The signal shown in Figure 1 indicates a variation in the stride interval with a standard deviation of 0.12 seconds, and the resolution of the measurement is of the order 0.01 seconds. What can we learn from a time series that has such a potentially substantial error? Suppose our time series consists of the superposition of two independent processes. One process is determined by the dynamics of the system and the other by measurement error, so that the second moment of the time series after *n *intervals is given by

<*X*(*t*)^2^> = *An *+ *Bn*^δ ^    (26)

The first process is, of course, that due to measurement error, modeled as a simple random walk, with strength *A*. For *δ *> 1 the second process is a persistent random walk and dominates for *n *> 1. In such a case we would expect for *n *sufficiently large, where the relative size of *A *and *B *determines what is meant by sufficiently large, to find the scaling

<*X*(λ*t*)^2^> ≈ λ^δ ^<*X*(*t*)^2^>.     (27)

This scaling was, in fact, observed for the data depicted in Figure 1, as well as for the other gait time series obtained in this study [7-9]. From the results of these earlier analyses we conclude that the level of statistical variation in the data, due to measurement error, will not change the conclusions drawn from subsequent analysis.

**Figure 2:**
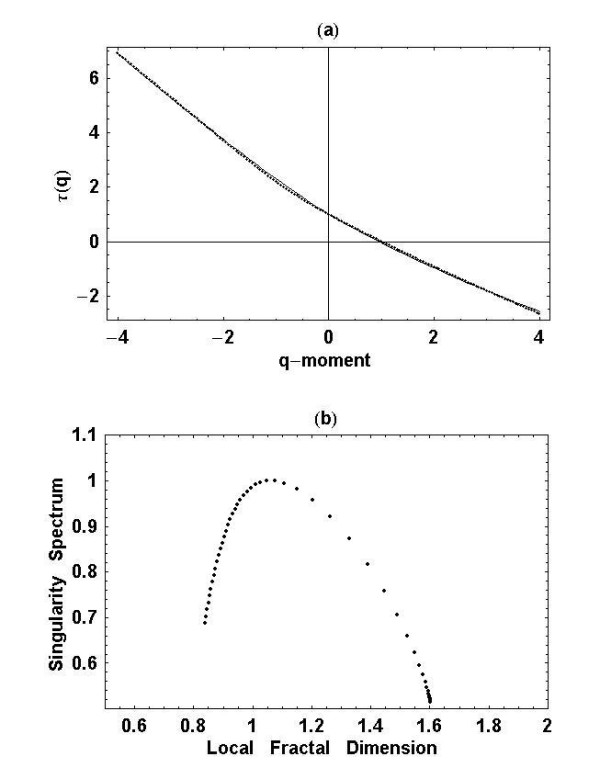
**Empirical mass exponent and singularity spectrum: **(a) The mass exponent is determined using the partition function from (28) and given by the dots for a typical data set. The solid curve is the quadradic least-squares fit of (29) to the calculated points. (b) The singularity spectrum is determined from the mass exponent using (9).

As mentioned above, a time series is monofractal when the mass exponent *τ(q) *is linear in *q*, otherwise the underlying process is multifractal. We apply the partition function measure and numerically evaluate



and the results are depicted in Figure 2a. Rigorously, the expression for the mass exponent requires *δ *→ 0, but we cannot do that with the data, so there is some error in our results. The significance of that error is to be determined. In Figure 2a we only show the mass exponent for a typical walker from the ten subjects, since they individually do not look too different from the curve shown. It is clear from the figure that the mass exponent is not linear in the moment index *q*. In Table 1 we record the fitting coefficients for each of the ten time series using the fitting equation for the mass exponent suggested by the solution to the fractional Langevin equation,

**Table 1:** The fitting parameters for the mass exponent *τ(q) *are listed. The column-*a*_1 _is the fractal dimension for the time series. In each case the fractal dimension agrees with that obtained earlier using a different method [7]. The last two columns denote the Lévy index and the statistical significance of the comparison of the empirical and theoretical values is *p *= 0.01

Walker	-a_1_	a_2_	Empirical Lévy index	Theoretical Lévy index
1	1.26	0.13	1.57	1.52
2	1.41	0.19	1.57	1.82
3	1.32	0.09	1.83	1.64
4	1.26	0.24	1.54	1.52
5	1.12	0.28	1.47	1.24
6	1.07	0.07	1.84	1.14
7	1.17	0.07	1.69	1.34
8	1.29	0.27	1.39	1.58
9	1.14	0.12	1.63	1.28
10	1.17	0.12	1.64	1.34
Averages	1.21 ± 0.10	0.15 ± 0.07	1.61 ± 0.15	1.44 ± 0.21

τ(*q*) = 1 + *a*_1_*q *+ *a*_2_|*q*|^α^.     (29)

The fit to the data using (29) is indicated by the solid curve in Figure 2a.

The singularity spectrum can now be determined using the Legendre transformation by at least two different methods. One procedure is to use the fitting equation substituted into (9). We do not do this here, but we note in passing that if (29) is inserted into (8), the fractal dimension is determined by the *q = 0 *moment to be



The values of the parameter *a*_1 _listed in Table 1 agree with the fractal dimensions obtained earlier using a scaling argument for the same data [7].

A second method for determining the singularity spectrum, the one we use here, is to numerically determine both *τ(q) *and its derivative. In this way we calculate the multifractal spectrum directly from the data using (9). It is clear from Figure 2b that we obtain the canonical form of the spectrum, that is, *f(h) *is a convex function of the scaling parameter *h*. The peak of the spectrum is determined to be the fractal dimension, as it should. Here again we have an indication that the interstride interval time series describes a multifractal process, but we stress that we are only using the qualitative properties of the spectrum for *q > 0*, due to the sensitivity of the numerical method to weak singularities. This sensitivity is apparent from the asymmetry of the empirical singularity spectrum in Figure 2b. These results are in agreement with the weak multifractality found by Scafetta et al. [31] using a different interstride interval data set.

**Figure 3:**
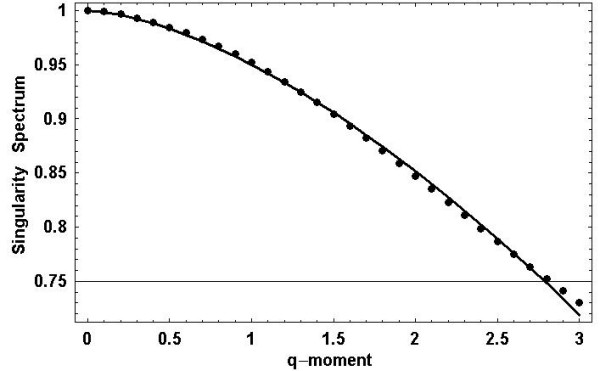
**Singularity spectum in terms of moments: **The singularity spectrum is calculated as a function of the moment-order and denoted by the dots using (9) for a typical data set. The solid curve is the least-squares fit of (29) to the calculated points.



It is clear from Figure 3 that the singularity spectrum calculated from the data for positive *q *are well fit by the solution to the fractional Langevin equation with the parameter values α = 1.57 and *a*_2 _= 0.13, obtained through a mean-square fit of (25) to the data points. Note that this fit to the scaling exponent is denoted as the empirical Lévy index in Table 1. Adjacent to this column is the theoretical Lévy index obtained from the relation

called the Lévy-walk diffusion relation [32] and which relates the scaling exponents when the underlying statistical process is an α-stable Lévy statistical process. Note that the Lévy probability density *p(x, t) *satisfies the scaling relation [32]



A comparison of the two columns for the Lévy index in Table 1, empirical and theoretical, using a statistical *t*-test, indicates statistical significance at the *p *= 0.01 level.

## Conclusion

The nonlinear form of the mass exponent *τ(q) *in Figure 2a, the convex form of the singularity spectrum *f(h) *in Figure 2b and the fit to *f(q) *in Figure 3, are all indications that interstride interval time series are multifractal. This analysis is further supported by the fact that the maxima of the singularity spectra coincide with the fractal dimensions determined previously using the scaling properties of the time series without the construction of a random walk trajectory [7]. A complete discussion of the limitations associated with determining the multifractal nature of interstride intervals using the singularity spectrum with limited data is given by Scafetta et al [31]. Furthermore, the empirical values of the Lévy index in Table 1 are consistent with those predicted using Lévy-walk diffusion relation [32] at the 0.01 level of significance.

It has been suggested that the CPG for gait consists of a random walk among a number of neural centers, thereby giving rise to its fractal behavior [5,6]. This model gives rise to a process having Gauss statistics and a long-time memory determined by the scaling index. The present results, however, point in a different direction. Recall that anamolous diffusion (δ ≠ 1) can arise in two distinct ways. The more familiar is that of a random walk with memory, in which the statistics are Gaussian, but the frequency spectrum is given by *P*(ω) ∝ 1/ω^δ-1^. The second way anomalous diffusion can arise is through the scaling of the probability density as given by (32). If the statistics are Gaussian then the scaling indices are related by δ = 2 μ and for ordinary diffusion μ = 1/2 impling δ = 1; in addition, for μ ≠ 1/2 the process is that of fractional Brownian motion. However, when the statistics are Lévy stable the second moment diverges and special methods must be employed to obtain second-moment scaling.

Shlesinger et al.[33] showed that when the steps in a random walk can be arbitrarily long and the length of time required to take a step is accounted for in the walking process, one obtains a Lévy diffusion process with a finite second moment. The second moment in such a Lévy-walk has a scaling index given by (31) with *δ = 1/α*. Consequently, the quality of the fit of the Lévy index obtained using the Lévy-walk diffusion relation to that obtained from the singularity spectrum, given by the solution to the fractional Langevin equation, suggests that the scaling in the interstride interval data may not be due solely to long-term memory, as previous investigators have concluded. Instead the observed scaling in interstride interval time series might be due to both long-time memory and statistics.

We use the fractional Langevin equation to describe the motor control process rather than the random walks of previous authors because of the direct correspondence between the microscopic dynamics and the macroscopic fractional derivatives established by Grigolini et al. [34]. The latter authors demonstrate that the existence of a clear separation between microscopic and macroscopic time scales supports the use of random walks and traditional statistical mechanics to model the phenomena of interest. This separation of time scales would be consistent with the traditional random walk way of modeling memory in CPG. However, when the microscopic time scales diverge, such that they overlap with the macroscopic time scale, ordinary statistical mechanics breaks down and the non-differentiabiltiy of the microscopic dynamics is transmitted from the microscopic to the macroscopic level in the form of fractional derivatives. In the present context a manifestation of an inverse power-law distribution of neuron firing would be a fractional differential equation of motion for motor response.

Stated somewhat differently, Grigolini et al [34] showed that the fractional derivative in the fractional Langevin equation can be interpreted in terms of an inverse power-law waiting time distribution function using a Continuous Time Random Walk Model. Thus, not only is the frequency accessed by the control system selected randomly, but the length of time it spends at that particular frequency in SCPG is also random. This waiting time distribution function is inverse power law and directly proportional to the fractional integral kernel. The fractional Langevin equation implies this full dynamical picture and appears to be consistent with the human gait data.

We are cognizant of the fact that to establish that the scaling observed in interstride interval data is due to statistics and memory, rather than long-time memory alone, requires more than the limited analysis presented here. So we put this speculation in the form of a hypothesis which we are presently testing using extensive interstride interval data available from *Physionet*. The results of these tests will be presented elsewhere.

## Supplementary Material

Additional File 1 List of symbols used. Click here for file

## References

[B1] Collins JJ, Richmond SA (1994). Hard-wired central pattern generators for quadrupedal locomotion. Biological-Cybernetics.

[B2] Cohen AH, Rossignol S, Grillner S (1988). Neural control of rhythmic movements in vertebrates.

[B3] Collins JJ, Stewart IN (1993). Coupled nonlinear oscillators and the the symmetries of animal gait. J Nonlinear Sci.

[B4] West BJ, Scafetta N (2003). Nonlinear dynamical model of human gait. Phys Rev E.

[B5] Hausdorff JM, Peng CK, Ladin Z, Wei JY, Goldberger AL (1995). Is walking a random walk? Evidence for long-range correlations in stride interval of human gait. J Appl Physiol.

[B6] Hausdorff JM, Mitchell SL, Firtion R, Peng CK, Cudkowicz ME, Wei JY, Goldberger AL (1997). Altered fractal dynamics of gait: reduced stride-interval correlations with aging and Huntington's disease. J Appl Physiol.

[B7] Griffin L, West DJ, West BJ (2000). Random stride intervals with memory. J Biol Phys.

[B8] West BJ, Griffin L (1998). Allometric control of human gait. Fractals.

[B9] West BJ, Griffin L (1999). Allometric control, inverse power laws and human gait. Chaos, Solitons & Fractals.

[B10] West BJ, Zhang R, Sanders AW, Miniyar S, Zuckerman JH, Levine BD (1999). Fractal fluctuations in transcranial Doppler signals. Physical Review E.

[B11] Peng CK, Mietus JE, Hausdorff JM, Havlin S, Stanley HE, Goldberger AL (1993). Long-range anticorrelations and non-Gaussian behavior of the heartdeat. Phys Rev Lett.

[B12] West BJ (1999). Physiology, Promiscuity and Prophecy at the Millennium: A Tale of Tails.

[B13] Szeto HH, Cheng PY, Decena JA, Cheng Y, Wu DL, Dwyer G (1992). Fractal properties in fetal breathing dynamics. Am J Physiol.

[B14] Ivanov PC, Amaral LA, Goldberger AL, Havlin S, Rosenblum MG, Stanley HE, Struzik ZR (1999). Multifractality in human heartbeat dynamics. Nature.

[B15] Montroll EW, West BJ, Montroll EW, Lebowitz JL (1987). An Enriched Collection of Stochastic Processes. Fluctuation phenomena, Pbk ed, updated edn.

[B16] Weiss GH (1994). Aspects and applications of the random walk.

[B17] Falconer KJ (1990). Fractal geometry mathematical foundations and applications.

[B18] Muzy JF, Bacry E, Arneodo A (1991). Wavelets and multifractal formalism for singular signals: Application to turbulence data. Physical Review Letters.

[B19] Muzy JF, Bacry E, Arneodo A (1993). Multifractal formalism for fractal signals: The structure-function approach versus the wavelet-transform modulus-maxima method. Phyical Review E.

[B20] Castaign B, Gagne Y, Hopfinger E (1990). Velocity probability density-functions of high Reynolds-number turbulence. Physica D.

[B21] Mallat SG (1999). A Wavelet Tour of Signal Processing.

[B22] Ashkenazy Y, Hausdorff JM, Ivanov PCh, Goldberger AL, Stanley HE (2002). A stochastic model of human gait dynamics. Physica A.

[B23] Schmitt FG, Seuront L (2001). Multifractal random walk in Copepod behavior. Physica A.

[B24] Feder J (1988). Fractals.

[B25] Feller W (1967). An introduction to probability theory and its applications.

[B26] Schertzer D, Lovejoy S, Schmit F, Chiguirinskaya Y, Marsan D (2005). Multifractal cascade dynamics and turbulent intermittency. Fractals.

[B27] Lindenberg K, West BJ (1990). The nonequilibrium statistical mechanics of open and closed systems.

[B28] West BJ, Grigolini P, Bologna M (2004). Physics of Fractal Operators.

[B29] Bassigthwaighte B, Liebowitch LS, West BJ (1994). Fractal Physiology.

[B30] Rajagopalan B, Tarboton DG (1993). Understanding complexity in the structure of rainfall. Fractals.

[B31] Scafetta N, Griffin L, West BJ (2003). Holder exponent spectra for human gait. Physica A.

[B32] Scafetta N, West BJ (2004). Mutiscaling comparative analysis on "earthquake conversations" in California:. Phys Rev Lett.

[B33] Shlesinger MF, West BJ, Klafter J (1987). Lévy dynamics for enhanced diffusion: an application to turbulence. Phys Rev Lett.

[B34] Grigolini P, Rocco A, West BJ (1999). Fractional calculus as a macroscopic manifestation of randomness. Phys Rev E.

