# Supplementary material for: Fractional Langevin model of gait variability
Source: J Neuroeng Rehabil. 2005 Aug 2;2:24. doi: 10.1186/1743-0003-2-24 (PMC1224863; doi:10.1186/1743-0003-2-24)
Supplement: Additional File 1 — List of symbols used. [file 1743-0003-2-24-S1.doc]

### List of symbols used

|  | Average over an ensemble of realization of z-variable |
| --- | --- |
|  | q^th^ moment of *x* |
| *h* | local fractal exponent |
| *μ* | probability measure |
| *S_q_* | partition function |
| *T(n,δ)* | random walk trajectory at n^th^ step and cell size δ |
| *D(q)* | generalized dimension of q^th^ moment |
| *D, D(0)* | fractal dimension |
| *τ(q)* | mass exponent |
| *ζ(q)* | structure function exponent |
| *f(h), f(q)* | singularity spectrum in local fractal exponent *h* or *q* |
| *X(t)* | stochastic system variable |
| *ξ(t)* | random noise; Wiener process |
| *K(t)* | memory kernel |
| *D^β^_t_[.]* | Riemann-Liouville fractional derivative |
| *D^-β^_t_[.]* | Riemann-Liouville fractional integral |
| *K_β_(t)* | kernel for fractional integral |
|  | Probability density of phase space variable *x* and time *t* |
| *δ* | scaling exponent for second moment |
| *α* | Lévy index |
